# Presentation of a 32-Year-Old Female Patient With Rapidly Growing Oral Squamous Cell Carcinoma: Report of a Rare Case

**DOI:** 10.7759/cureus.41042

**Published:** 2023-06-27

**Authors:** Monal M Kukde, Ashish Lanjekar, Komal Deotale, Obaid Noman, Deepak Selokar

**Affiliations:** 1 Dentistry, Datta Meghe Medical College, Datta Meghe Institute of Higher Education & Research, Wardha, IND; 2 Oral Medicine and Radiology, Swargiya Dadasaheb Kalmegh Smruti Dental College and Hospital, Nagpur, IND; 3 Oral Medicine and Radiology, Swargiya Dadasaheb Kalmegh Smruti Dental College And Hospital, Nagpur, IND; 4 Pathology, Jawaharlal Nehru Medical College, Datta Meghe Institute of Higher Education & Research, Wardha, IND; 5 Preventive Medicine, Public Health Department Zilla Parishad, Nagpur, IND

**Keywords:** early diagnosis, tobacco, carcinoma, alcohol, oral squamous cell carcinoma

## Abstract

In this case report, a 32-year-old female patient from Central India was reported. Her primary complaint was pain and swelling in the lower left back region of her jaw, which had been present for one month. On extraoral examination, asymmetrical facial symmetry was observed, and a 3 cm swelling was present on the left side of her face. An intraoral examination showed a reddish-pink ulceroproliferative growth on the left buccal mucosa. Under local anesthesia, an incisional biopsy was performed. A conclusive diagnosis of well-differentiated squamous cell carcinoma affecting the left buccal mucosa was made based on clinical and histopathological testing. The patient was scheduled for a follow-up appointment after being referred to the Cancer Institute for the required treatment. After three months, the patient reported to the department with extensive swelling on the left side of the face, measuring 6 cm in diameter. On intraoral examination, a more restricted mouth opening compared to the previous examination. The ulceroproliferative growth was firm and tender and exhibited continuous bleeding. As the patient delayed seeking proper care, the condition became more aggressive, and she lost her life. This case of oral squamous cell carcinoma (OSCC) in a 32-year-old female patient is rare, as men are more commonly affected by OSCC in Central India due to their higher prevalence of unhealthy practices. This case highlights the rapid progression of the disease if appropriate treatment is not initiated promptly upon diagnosis.

## Introduction

Oral squamous cell carcinoma (OSCC) accounts for over 90% of oral cancers. The eighth most common cause of cancer-related fatalities worldwide is OSCC [1]. As per the GLOBOCAN 2018 report, there are around 354,864 newly diagnosed cases of lip and oral cavity cancer per year, which accounts for about 2% and 0.5% of all malignancies. India, Pakistan, Bangladesh, Taiwan, and Sri Lanka are developing nations with high incidence rates. Smoking and tobacco chewing are the two etiologic variables that are significant for the development of OSCC [2]. The OSCC-originating cell is the oral keratinocyte [3]. The lips, gingiva, palate, and dorsum of the tongue are common sites of OSCC [4]. Clinically, it appears as a broad exophytic mass with a verrucous, pebbled, or generally smooth surface texture and develops into a necrotic ulcer with uneven, elevated, and indurated edges. OSCC bleeds easily and becomes uncomfortable when it has an oral secondary infection. Giant lesions may obstruct normal swallowing, mastication, or speech. If extracapsular spread occurs into the nearby connective tissue, the afflicted lymph nodes, which are stiff and nontender, will become matted and fixed [5]. Tumor, nodes, and metastases (TNM) staging correlates with survival rates. The surgical procedure includes maxillectomy, mandibulectomy, glossectomy, radical neck dissection, and Mohs surgery. Chemoradiotherapy is used in inoperable cases. Various other modalities include photodynamic therapy, nanocarrier-based drug delivery technology, etc. Medicinal treatment includes antibiotics, analgesics, epidermal growth factor receptor (EGFR) inhibitors such as nimotuzumab and cetuximab, and cyclooxygenase 2 (COX-2) inhibitors such as nimesulide [6]. The purpose of this case report is to highlight the rarity of OSCC in a young woman and explore the etiological and differential diagnosis elements associated with this disease in this particular age group.

## Case presentation

**Figure 1:**
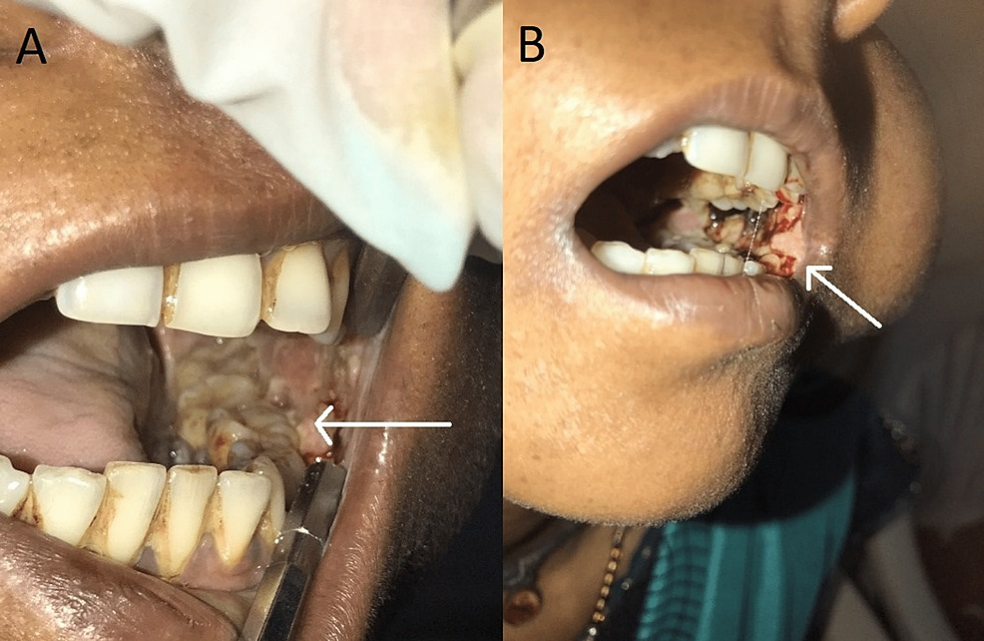
Extent of ulcerative proliferative growth on left buccal mucosa: (A) first visit and (B) second visit.

A 32-year-old female patient reported to the Department of Dentistry of Shalinitai Meghe Hospital and Research Centre, Nagpur, and diagnostic procedures were performed in the Department of Oral Medicine and Radiology (Swargiya Dadasaheb Kalmegh Smruti Dental College and Hospital Nagpur). The patient had a one-month-old primary complaint of discomfort and swelling in the lower left back area of the jaw. The pain was severe, continuous, and aggravated by mastication and speech. There was no relevant medical or dental history. The patient chewed tobacco six to seven times daily for the last eight years. Her face was asymmetric during the extraoral evaluation with extensive swelling on the left side (3 cm in diameter), extending superoinferiorly (S/I) from the infraorbital region to the lower border of the mandible and anteroposteriorly (A/P) from the left corner of the mouth to the angle of the mandible. The color was the same as that of the adjacent skin. All findings were confirmed on palpation; the swelling was febrile, soft, and tender. The right and left submandibular lymph nodes and the submental lymph nodes were tender and mobile. There was a restricted mouth opening (25 mm) on the intraoral examination. A reddish-pink ulceroproliferative growth of size 2 cm was seen in the left buccal mucosa, extending A/P from the mesial of tooth 36 to the distal of tooth 37 and S/I from the line of occlusion of the lower molars till the lower vestibule (Figure 1).

**Figure 2:**
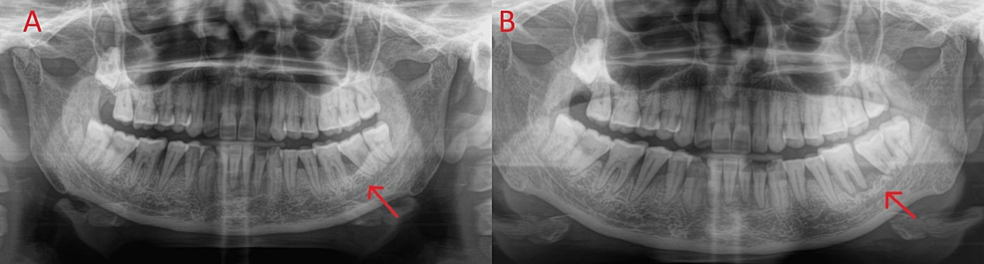
Orthopantomogram showing bone loss: (A) first visit and (B) second visit.

The shape of the lesion was irregular, and the borders were raised. The lesion was nonscrapable, soft, and tender, and spontaneous bleeding was also witnessed. There was generalized melanin pigmentation, erythema, and blanching in the right and left buccal mucosa and the hard palate. A whitish patch was also evident in the right buccal mucosa, extending A/P from the right corner of the mouth to the mesial of teeth 14 and 44 and S/I from the upper vestibule to the lower vestibule. The lesion was nonscrapable and nontender. The vertical fibrous bands were palpable in both the mucosae. An orthopantomogram (OPG) revealed horizontal bone loss with teeth 34, 35, 36, 37, 46, and 47, indicative of (S/O) moderate periodontitis. Vertical bone loss was observed with teeth 45, 42, 41, 31, and 32, indicating severe periodontitis (Figure 2).

**Figure 3:**
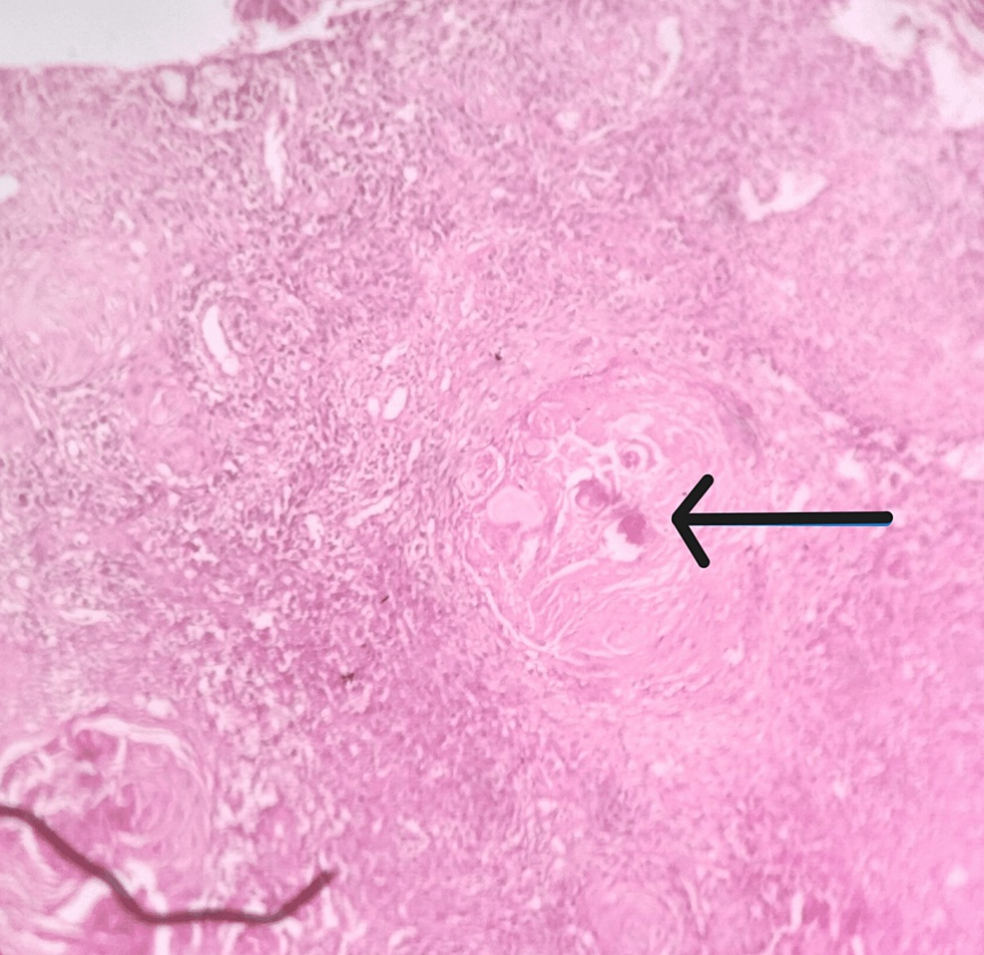
Histopathlogy image showing keratin pearls (the arrow shows keratin pearls).

The patient was advised of blood investigations. Her complete blood count (CBC), Hepatitis B surface antigen (HBsAg), and hemoglobin A1c (HbA1c) levels were within normal limits, and the HIV test was nonreactive. Under local anesthesia, an incisional biopsy was performed, and histopathological analysis was conducted. The specimen revealed stratified squamous epithelium of the orthokeratinized type, dysplastic epithelial cells infiltrating the connective tissue, and numerous keratin pearls. Finally, a conclusive diagnosis of well-differentiated squamous cell carcinoma involving the left buccal mucosa was established (Figure 3).

The patient underwent counseling; received a 15-day prescription for antibiotics, analgesics, antacids, antioxidants, and betadine mouthwash; was referred to a cancer Institute (National Cancer Institute, Nagpur, India) with all respective reports and radiographs; and was recalled after 15 days for follow-up. After 15 days, the patient did not appear in the department or return calls for follow-up care. On August 17, 2022, the patient reported back to the department with a primary complaint of severe discomfort and significant swelling in the lower left back region of the jaw. Swelling extended A/P from the left corner of the mouth to the angle of the mandible and S/I from the infraorbital area to the lower border of the mandible. The size was 6 cm in diameter. The color was reddish with a shiny surface. All findings were confirmed on palpation; the swelling was febrile, firm, and tender. Both the right and left submandibular lymph nodes and the submental lymph nodes were tender and mobile. On intraoral examination, there was a more restricted mouth opening (16 mm) than earlier. The extent of ulceroproliferative growth was A/P from the left corner of the mouth to the retromolar area and S/I from the upper vestibule to the lower vestibule. The shape was irregular, and the borders were raised. The lesion was not scrapable, was firm, and tender, and there was continuous bleeding. Other intraoral findings were identical to those mentioned above. The patient was again advised for OPG. It revealed more extensive vertical bone loss with teeth 36 and 37, along with furcation involvement S/O severe periodontitis with teeth 36 and 37. The patient was again given in-depth counseling and was directed to the National Cancer Institute. The patient again discontinued follow-up and did not undergo any treatment at the National Cancer Institute. Due to the aggressive nature of condition, the patient's health deteriorated, ultimately resulting in her demise.

## Discussion

OSCC is a growing concern worldwide. The International Association of Cancer Registries (IACR) initiated the GLOBOCAN project, which aims to coordinate and provide a comprehensive global overview of various types of cancers, including oral cancer. The worldwide projected age-standardized incidence rate of OSCC is four per 100,000 [7]. Only 6% of OSCC cases are evident in people aged under 40 years. Young patients with OSCC are arbitrary. The average instances of young OSCC bearers in the literature range from 30.8 to 34.2, with a greater male predilection [8]. Due to the increased exposure to carcinogenic chemicals, including alcohol and tobacco, as was in this case, a small number of studies had also demonstrated a female propensity for OSCC.

The primary risk factor for OSCC is believed to be tobacco use, both now and in the past. Large OSCC lesions can infiltrate multiple, continuous locations. Andisheh-Tadbir et al. demonstrated that the tongue and the buccal mucosa are the two most common locations for OSCC [9]. OSSC in males is predominantly poorly and moderately differentiated, while in females, it is primarily well and moderately differentiated, as observed in this case [10]. According to an epidemiological study of OSCC conducted in India by Chattopadhyay [11] and Mathew et al. [12], oral cancer is more common in younger men and women.

Oral keratinocytes are precursor cells of OSCC, and xenobiotic metabolizing enzymes (XME) influence the metabolism of chemicals that cause cancer [3]. The primary cause is DNA mutation, which is worsened by exposure to chemical, physical, or microbial mutagens. Clinically, OSCC presents as a broad-based exophytic mass with a verrucous, pebble-like, or generally smooth surface texture. It can also resemble verrucous leukoplakia or erythroleukoplakia and eventually develop into a necrotic ulcer with irregular, elevated, and indurated borders. With secondary infection, OSCC bleeds easily and becomes painful, as observed in this case. The affected lymph nodes are firm and nontender; if extracapsular spread into the surrounding connective tissue, they will be fixed and matted. The staging of TNM correlates with the survival rate. Other treatment options for OSCC include photodynamic therapy, nanocarrier-based medication delivery technology, surgical removal, and radiation therapy [5].

## Conclusions

We observed OSCC in a 32-year-old female patient with a major risk factor as betel nut chewing with tobacco. A high level of suspicion, early diagnosis, pathological diagnosis, and a multidisciplinary approach is required to treat these patients. If prompt and appropriate treatment is not initiated upon obtaining the diagnosis, OSCC can become more aggressive. Reducing risk factors such as heavy tobacco use, prolonged tobacco exposure, and early habit-building is required to reduce incidence rates of oral cancer. 
